# The Stomach Tube in Practical Medicine

**Published:** 1899-10-14

**Authors:** 


### THE STOMACH TUBE IN PRACTICAL MEDICINE.

In a clinical lecture on "Some Diseases of the Stomach and their Diagnosis by Chemical Methods," Professor Vaughan Harley[1] describes the methods in use for ascertaining the nature of the stomach contents, and the deductions which may legitimately be drawn from such observations. He says that it is now gradually becoming recognised that the majority of cases of so-called dyspepsia are undoubtedly of a neurotic origin, *i.e.*, if the term "neurotic" be carefully restricted to its exact meaning, and he also points out that, notwithstanding the ridicule cast, even by so-called leaders of the profession, upon the possibility of making a diagnosis in gastric affection by means of chemical analysis, it is as a fact possible to arrive at such a diagnosis by chemistry alone. The points in regard to stomach digestion, on which it is most important to obtain information by such methods, are: (1) The motor power of the stomach, its power of moving its contents and finally getting rid of them. In some cases of increased irritability, between three and four hours after a last meal of meat and bread, one may find the stomach empty, while in cases of delayed motility, without any pyloric obstruction, even 16 hours after taking food one may find a residue of the meal still remaining in the stomach. (2) The next question is as to the size of the stomach, and this is best determined by distending that viscus with gas. This may be done either by blowing air down the stomach tube or by giving the patient first half a teaspoonful of tartaric acid, and immediately following it half a teaspoonful of bicarbonate of soda, each dissolved in about a wineglass of water. One can then map out the general outline of the stomach. (3) By chemical tests applied to the stomach contents we ascertain in what direction the processes of digestion in a given case depart from the standard which may be accepted as representing the normal. The striking relation between excess or deficiency of hydrochloric acid and certain diseased conditions is well known; but Professor Harley shows that in ordinary cases of acid dyspepsia, as it is termed, the treatment much depends upon the character of the acid present. This condition, he says, must be divided into two groups—one the condition described as hyperchlorhydria, where the excess of acid is hydrochloric; and the other that in which the patient suffers from hyperacidity, in which the increase is in the volatile acids. Now, as showing the value of chemical examination of the stomach contents it is well to bear in mind how opposite are the lines of treatment which are found most useful in these two conditions, alike as they may seem, and in fact do seem, in the presence of an excess of acid. In the first condition, although by trying to diminish the acidity by alkalies we relieve the symptoms, still at the same time we must never lose sight of the fact that if this cure is not rapidly carried out it tends to increase the acids formed. Thus the treatment by bismuth and prussic acid, which is so valuable in early cases, is about the worst that can be employed, when by dieting, &c., the cause of the hypersecretion is not removed, for in these cases we mask the symptoms, and we tend slowly to get increased fermentation of the stomach, and, as a rule, a tendency to atonic dilatation. On the other hand, cases in which the hyperacidity is due to increase in the volatile acids are just those which are most benefited by taking bismuth and sodium carbonate some time after food. Chemical analysis, then, not only shows them to belong to a different division, but sets them apart as requiring a different treatment. In cases of ulcer of the stomach and of carcinoma, and as a guide to the treatment of the stomach in dyspepsias of neurotic origin, chemical analysis of the stomach contents is also of constant service; but these are matters with which we will deal later, only repeating here what Professor Vaughan Harley says at the beginning of his lecture, that "during the last 15 years our conception of diseases of the stomach has been entirely revolutionised."

[1] Practitioner, Oct., 1899.

